# Supplementary material for: Association of Sugar-Sweetened Beverage Consumption and Moderate-to-Vigorous Physical Activity with Childhood and Adolescent Overweight/Obesity: Findings from a Surveillance Project in Jiangsu Province of China
Source: Nutrients. 2023 Sep 27;15(19):4164. doi: 10.3390/nu15194164 (PMC10574764; doi:10.3390/nu15194164)
Supplement: Supplementary file 1 [file nutrients-15-04164-s001.zip › nutrients-2621701-supplementary.pdf]

Supplementary Material

Supplementary Figure S1. Proportion of children and adolescents with overweight/obesity in association with their SSB and MVPA characteristics by age and gender during 2021-2022 academic year

Supplementary Table S1. Characteristics of 119467 participants according to SSB consumption in Jiangsu Province, China

Supplementary Table S2. Characteristics of 119467 participants according to inadequate MVPA in Jiangsu Province, China

Supplementary Table S3. Characteristics of 119467 participants according to SSB-MVPA categories in Jiangsu Province, China

Supplementary Table S4. The association of the SSB consumption habit with overweight/obesity in total sample and by age/gender subgroups

Supplementary Table S5. The association of the MVPA level with overweight/obesity in total sample and by age/gender subgroups

Supplementary Table S6. The separate association of SSB consumption or inadequate MVPA with BMI in total sample and by age/gender subgroups

Supplementary Table S7. The joint association of SSB and MVPA with BMI among 119467 children and adolescents in Jiangsu Province, China

Supplementary Figure S2. Joint association of SSB and MVPA with BMI, stratified by age or gender

Supplementary Figure S1. Proportion of children and adolescents with overweight/obesity in association with their SSB and MVPA characteristics by age and gender during 2021-2022 academic year

(a)

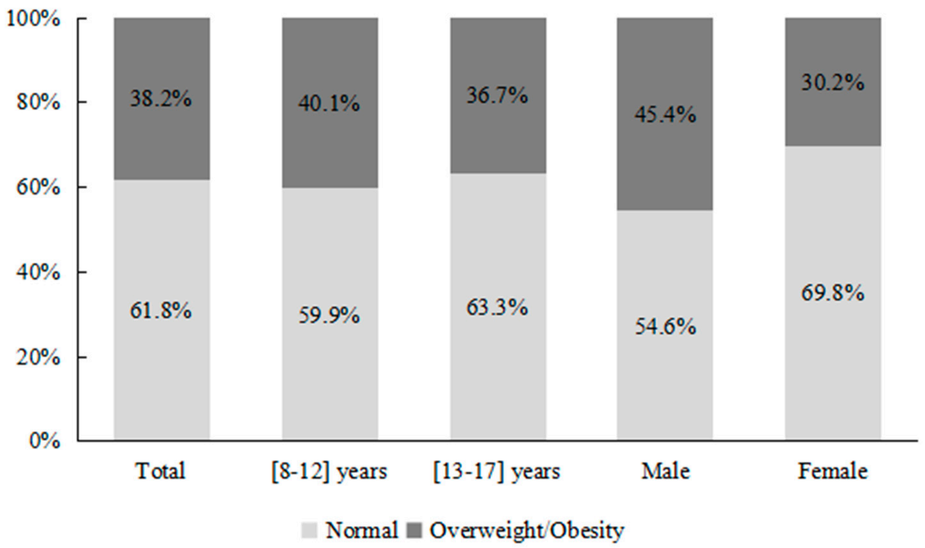

(b)

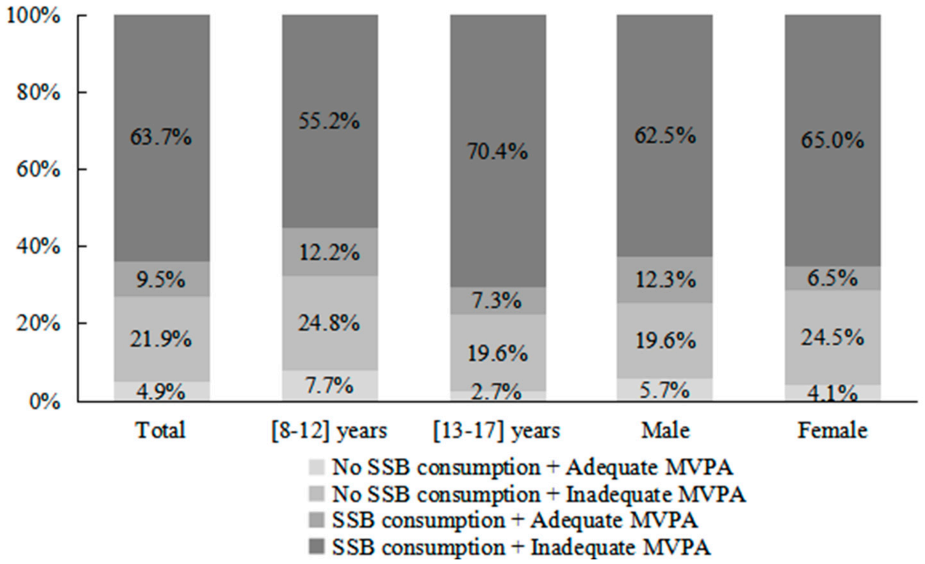

Note: the upper figure (a) shows the proportion of participants with and without overweight/obesity, in total sample and by age/gender subgroups; the lower figure (b) shows the proportion of participants across joint classifications of SSB and MVPA, in total sample and by age/gender subgroups.

Supplementary Table S1. Characteristics of 119,467 participants according to SSB consumption in Jiangsu Province, China

Variables	No SSB consumption (32,041)	SSB consumption (87,426)	p-value [a]
	N(%)		
Age group,year			
[8-12]	17,232(32.52)	35,764(67.48)	<0.001***
[13-17]	14,809(22.28)	51,662(77.72)	
Gender			
Male	15,837(25.23)	46,922(74.77)	<0.001***
Female	16,204(28.57)	40,504(71.43)	
Residence			
Urban	17,265(25.33)	50,903(74.67)	<0.001***
Rural	14,776(28.80)	36,523(71.20)	
Region of Jiangsu Province			
Southern	13,000(25.55)	37,887(74.45)	<0.001***
Central	4,894(28.25)	12,431(71.75)	
Northern	14,147(27.60)	37,108(72.40)	
Family types			
Core families	14,262(27.08)	38,410(72.92)	0.075
Others [b]	17,779(26.62)	49,016(73.38)	
Maternal education			
Primary or illiterate	3,326(23.75)	10,678(76.25)	<0.001***
Junior or senior high school	19,828(25.70)	57,330(74.30)	
College	7,747(30.85)	17,368(69.15)	
Post-graduate and beyond	1,140(35.74)	2,050(64.26)	
Paternal education			
Primary or illiterate	1,930(24.88)	5,826(75.12)	<0.001***
Junior or senior high school	20,430(25.30)	60,308(74.70)	
College	8,402(30.75)	18,921(69.25)	
Post-graduate and beyond	1,279(35.04)	2,371(64.96)	

[a] p-values were calculated from chi-square tests.

[b] Including extended families, one-parent families, re-married families, and inter-generational families.

*p<0.05, **p<0.01, ***p<0.001.

Supplementary Table S2. Characteristics of 119,467 participants according to inadequate MVPA in Jiangsu Province, China

Variables	Adequate MVPA (17,237)	Inadequate MVPA (102,230)	p-value [a]
	N(%)		
Age group,year			
[8-12]	10,589(19.98)	42,407(80.02)	<0.001***
[13-17]	6,648(10.00)	59,823(90.00)	
Gender			
Male	11,245(17.92)	51,514(82.08)	<0.001***
Female	5,992(10.57)	50,716(89.43)	
Residence			
Urban	9,268(13.60)	58,900(86.40)	<0.001***
Rural	7,969(15.53)	43,330(84.47)	
Region of Jiangsu Province			
Southern	7,373(14.49)	43,514(85.51)	0.635
Central	2,524(14.57)	14,801(85.43)	
Northern	7,340(14.32)	43,915(85.68)	
Family types			
Core families	7,176(13.62)	45,496(86.38)	<0.001***
Others [b]	10,061(15.06)	56,734(84.94)	
Maternal education			
Primary or illiterate	1,595(11.39)	12,409(88.61)	<0.001***
Junior or senior high school	10,204(13.22)	66,954(86.78)	
College	4,438(17.67)	20,677(82.33)	
Post-graduate and beyond	1,000(31.35)	2,190(68.65)	
Paternal education			
Primary or illiterate	928(11.96)	6,828(88.04)	<0.001***
Junior or senior high school	10,639(13.18)	70,099(86.82)	
College	4,593(16.81)	22,730(83.19)	
Post-graduate and beyond	1,077(29.51)	2,573(70.49)	

[a] p-values were calculated from chi-square tests.

[b] Including extended families, one-parent families, re-married families, and inter-generational families.

*p<0.05, **p<0.01, ***p<0.001.

Supplementary Table S3. Characteristics of 119,467 participants according to SSB-MVPA categories in Jiangsu Province, China

Variables	No SSB consumption +Adequate MVPA (5,877)	No SSB consumption +Inadequate MVPA (26,164)	SSB consumption+ Adequate MVPA (11,360)	SSB consumption+ Inadequate MVPA (76,066)	p-value [a]
	N(%)				
Age group,year					
[8-12]	4,100(7.74)	13,132(24.78)	6,489(12.24)	29,275(55.24)	<0.001***
[13-17]	1,777(2.67)	13,032(19.61)	4,871(7.33)	46,791(70.39)	
Gender					
Male	3,546(5.65)	12,291(19.58)	7,699(12.27)	39,223(62.50)	<0.001***
Female	2,331(4.11)	13,873(24.46)	3,661(6.46)	36,843(64.97)	
Residence					
Urban	3,012(4.42)	14,253(20.91)	6,256(9.18)	44,647(65.50)	<0.001***
Rural	2,865(5.58)	11,911(23.22)	5,104(9.95)	31,419(61.25)	
Region of Jiangsu Province					
Southern	2,407(4.73)	10,593(20.82)	4,966(9.76)	32,921(64.69)	<0.001***
Central	1,002(5.78)	3,892(22.46)	1,522(8.78)	10,909(62.97)	
Northern	2,468(4.82)	11,679(22.79)	4,872(9.51)	32,236(62.89)	
Family types					
Core families	2,461(4.67)	11,801(22.40)	4,715(8.95)	33,695(63.97)	<0.001***
Others [b]	3,416(5.11)	14,363(21.50)	6,645(9.95)	42,371(63.43)	
Maternal education					
Primary or illiterate	444(3.17)	2,882(20.58)	1,151(8.22)	9,527(68.03)	<0.001***
Junior or senior high school	3,249(4.21)	16,579(21.49)	6,955(9.01)	50,375(65.29)	

College	1,727(6.88)	6,020(23.97)	2,711(10.79)	14,657(58.36)	
Post-graduate and beyond	457(14.33)	683(21.41)	543(17.02)	1,507(47.24)	
Paternal education					
Primary or illiterate	273(3.52)	1,657(21.36)	655(8.45)	5,171(66.67)	<0.001***
Junior or senior high school	3,336(4.13)	17,094(21.17)	7,303(9.05)	53,005(65.65)	
College	1,780(6.51)	6,622(24.24)	2,813(10.30)	16,108(58.95)	
Post-graduate and beyond	488(13.37)	791(21.67)	589(16.14)	1,782(48.82)	

[a] p-values were calculated from chi-square tests.

[b] Including extended families, one-parent families, re-married families, and inter-generational families.

*$p<0.05$, **$p<0.01$, ***$p<0.001$.

Supplementary Table S4. The association of the SSB consumption habit with overweight/obesity in total sample and by age/gender subgroups

SSB Consumption		Total	By Age Group			By Gender		
		OR (95% CI)	[8-12] OR (95% CI)	[13-17] OR (95% CI)	P for interaction[d]	Male OR (95% CI)	Female OR (95% CI)	P for interaction[d]
SSB consumption (three categories, reference group is "never")								
Model 1[a]	sometimes	1.05 (1.02,1.08)**	1.02 (0.99,1.06)	1.07 (1.03,1.11)**	0.035*	1.01 (0.98,1.05)	1.09 (1.05,1.13)***	0.001**
	always	1.02 (0.97,1.07)	1.06 (0.97,1.15)	1.01 (0.94,1.07)		0.99 (0.93,1.05)	1.05 (0.97,1.14)	
Model 2[b]	sometimes	1.05 (1.02,1.08)**	1.02 (0.99,1.06)	1.07 (1.03,1.11)**	0.037*	1.01 (0.98,1.05)	1.09 (1.05,1.14)***	0.001**
	always	1.02 (0.97,1.07)	1.06 (0.98,1.15)	1.01 (0.95,1.07)		0.99 (0.93,1.06)	1.05 (0.97,1.14)	
Model 3[c]	sometimes	1.05 (1.02,1.08)***	1.02 (0.99,1.06)	1.07 (1.03,1.11)**	0.036*	1.02 (0.98,1.05)	1.09 (1.05,1.14)***	0.001**
	always	1.01 (0.96,1.06)	1.06 (0.97,1.15)	1.00 (0.94,1.07)		0.99 (0.93,1.05)	1.05 (0.97,1.14)	

[a] Model 1 adjusted for age and gender.

[b] Model 2 adjusted for age, gender, residence, regions, family types, paternal education, and maternal education.

[c] Model 3 adjusted for age, gender, residence, regions, family types, paternal education, maternal education, and MVPA.

[d] P for interaction was calculated using the likelihood ratio test by comparing the two models with and without the interactive term.

*p<0.05, **p<0.01, ***p<0.001; Abbreviations: OR, odds ratio; CI, confidence interval.

Supplementary Table S5. The association of the MVPA level with overweight/obesity in total sample and by age/gender subgroups

MVPA		Total	By Age Group			By Gender		
			[8-12]	[13-17]	P for	Male	Female	P for
		OR (95% CI)	OR (95% CI)	OR (95% CI)	interaction[d]	OR (95% CI)	OR (95% CI)	interaction[d]
MVPA (three categories, reference group is "high frequency ")								
Model 1[a]	moderate frequency	1.10 (1.06,1.13)***	1.12 (1.07,1.17)***	1.07 (1.02,1.13)**	0.472	1.14 (1.09,1.19)***	1.02 (0.97,1.08)	0.079
	low frequency	1.09 (1.05,1.13)***	1.09 (1.04,1.14)***	1.08 (1.02,1.13)**		1.15 (1.10,1.20)***	0.99 (0.94,1.05)	
Model 2[b]	moderate frequency	1.10 (1.06,1.13)***	1.12 (1.07,1.17)***	1.07 (1.02,1.13)**	0.544	1.14 (1.09,1.19)***	1.03 (0.97,1.09)	0.091
	low frequency	1.09 (1.05,1.12)***	1.09 (1.04,1.14)***	1.07 (1.02,1.13)**		1.15 (1.10,1.20)***	0.98 (0.92,1.03)	
Model 3[c]	moderate frequency	1.09 (1.06,1.13)***	1.12 (1.07,1.17)***	1.07 (1.01,1.13)*	0.538	1.14 (1.09,1.19)***	1.02 (0.96,1.08)	0.084
	low frequency	1.08 (1.05,1.12)***	1.08 (1.03,1.14)***	1.07 (1.02,1.13)***		1.15 (1.10,1.20)***	0.97 (0.92,1.03)	

[a] Model 1 adjusted for age and gender.

[b] Model 2 adjusted for age, gender, residence, regions, family types, paternal education, and maternal education.

[c] Model 3 adjusted for age, gender, residence, regions, family types, paternal education, maternal education, and SSB.

[d] P for interaction was calculated using the likelihood ratio test by comparing the two models with and without the interactive term.

*p<0.05, **p<0.01, ***p<0.001; Abbreviations: OR, odds ratio; CI, confidence interval.

Supplementary Table S6. The separate association of SSB consumption or inadequate MVPA with BMI in total sample and by age/gender subgroups

Variables	Total	By Age Group			By Gender		
	β (95% CI)	[8-12] β (95% CI)	[13-17] β (95% CI)	P for interaction[d]	Male β (95% CI)	Female β (95% CI)	P for interaction[d]
SSB Consumption (yes vs. no)							
Model 1[a]	0.22 (0.17,0.27)***	0.17 (0.10,0.25)***	0.26 (0.18,0.34)***	0.242	0.15 (0.07,0.23)***	0.29 (0.22,0.36)***	<0.001***
Model 2[b]	0.21 (0.15,0.26)***	0.15 (0.08,0.23)***	0.25 (0.17,0.33)***	0.220	0.14 (0.06,0.22)***	0.27 (0.20,0.34)***	<0.001***
Model 3[c]	0.21 (0.15,0.26)***	0.16 (0.08,0.23)***	0.25 (0.17,0.33)***	0.208	0.14 (0.06,0.22)***	0.27 (0.20,0.34)***	<0.001***
Inadequate MVPA (yes vs. no)							
Model 1[a]	0.37 (0.30,0.44)***	0.19 (0.07,0.31)**	0.43 (0.34,0.51)***	0.001**	0.46 (0.35,0.57)***	0.26 (0.17,0.35)***	0.125
Model 2[b]	0.34 (0.28,0.42)***	0.16 (0.04,0.28)**	0.40 (0.32,0.49)***	0.001**	0.45 (0.34,0.56)***	0.23 (0.14,0.31)***	0.125
Model 3[c]	0.35 (0.28,0.42)***	0.16 (0.04,0.28)**	0.41 (0.32,0.49)***	0.001**	0.45 (0.34,0.56)***	0.23 (0.14,0.32)***	0.115

[a] Model 1 adjusted for age and gender.

[b] Model 2 adjusted for age, gender, residence, regions, family types, paternal education, and maternal education.

[c] Model 3 adjusted for age, gender, residence, regions, family types, paternal education, maternal education, and SSB (when the main independent variable of interest was MVPA) or MVPA (when the main independent variable of interest was SSB).

[d] P for interaction was calculated using the likelihood ratio test by comparing the two models with and without the interactive term.

*p<0.05, **p<0.01, ***p<0.001; Abbreviations: CI, confidence interval.

Supplementary Table S7. The joint association of SSB and MVPA with BMI among 119,467 children and adolescents in Jiangsu Province, China

Groups	Model 1[a]		Model 2[b]	
	β (95% CI)	P value	β (95% CI)	P value
(A) No SSB consumption + Adequate MVPA	Ref		Ref	
(B) No SSB consumption + Inadequate MVPA	0.18(0.05,0.32)	0.008**	0.16(0.02,0.30)	0.020*
(C) SSB consumption + Adequate MVPA	0.19(0.13,0.24)	<0.001***	0.17(0.11,0.23)	<0.001***
(D) SSB consumption + Inadequate MVPA	0.62(0.53,0.71)	<0.001***	0.59(0.50,0.68)	<0.001***

[a] Model 1 adjusted for age and gender.

[b] Model 2 adjusted for age, gender, residence, regions, family types, paternal education, and maternal education.

*p<0.05, **p<0.01, ***p<0.001; Abbreviations: OR, odds ratio; CI, confidence interval.

Supplementary Figure S2. Joint association of SSB and MVPA with BMI, stratified by age or gender

(a) Subgroup analysis by age group

Subgroup	Coefficient (95% CI)	P value	P interaction
Age group [8-12]			
(A) No SSB consumption + Adequate MVPA	0.00		
(B) No SSB consumption + Inadequate MVPA	0.01(-0.20,0.22)	0.937	
(C) SSB consumption + Adequate MVPA	0.14(0.06,0.21)	<0.001***	
(D) SSB consumption + Inadequate MVPA	0.37(0.22,0.53)	<0.001***	
Age group [13-17]			0.025*
(A) No SSB consumption + Adequate MVPA	0.00		
(B) No SSB consumption + Inadequate MVPA	0.24(0.06,0.42)	0.009**	
(C) SSB consumption + Adequate MVPA	0.21(0.12,0.30)	<0.001***	
(D) SSB consumption + Inadequate MVPA	0.66(0.55,0.78)	<0.001***	

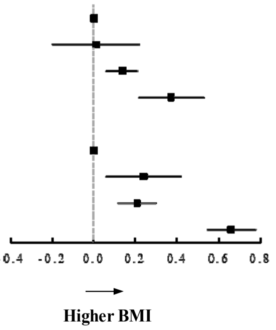

Note: P for interaction was calculated using the likelihood ratio test; *p<0.05, **p<0.01, ***p<0.001

(b) Subgroup analysis by gender

Subgroup	Coefficient (95% CI)	P value	P interaction
Male			
(A) No SSB consumption + Adequate MVPA	0.00		
(B) No SSB consumption + Inadequate MVPA	0.14(-0.08,0.35)	0211	
(C) SSB consumption + Adequate MVPA	0.09(0.00,0.18)	0.042*	
(D) SSB consumption + Inadequate MVPA	0.65(0.51,0.79)	<0.001***	
Female			<0.001***
(A) No SSB consumption + Adequate MVPA	0.00		
(B) No SSB consumption + Inadequate MVPA	0.17(0.00,0.34)	0.048*	
(C) SSB consumption + Adequate MVPA	0.26(0.18,0.34)	<0.001***	
(D) SSB consumption + Inadequate MVPA	0.51(0.40,0.63)	<0.001***	

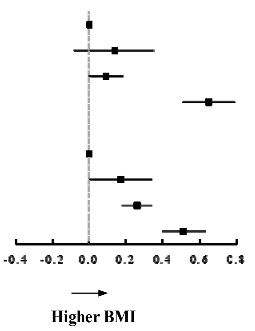

Note: P for interaction was calculated using the likelihood ratio test; *p<0.05, **p<0.01, ***p<0.001
